# Integrin β3 promotes cardiomyocyte proliferation and attenuates hypoxia-induced apoptosis via regulating the PTEN/Akt/mTOR and ERK1/2 pathways

**DOI:** 10.7150/ijbs.39414

**Published:** 2020-01-14

**Authors:** Lijiang Wei, Qingqing Zhou, Hua Tian, Yifan Su, Guo-hui Fu, Ting Sun

**Affiliations:** 1 Department of Cardiology, Shanghai Ninth People's Hospital, Shanghai Jiaotong University School of Medicine. Shanghai, 200025, China; 2 State Key Laboratory of Oncogenes and Related Genes, Shanghai Cancer Institute, Renji Hospital, Shanghai Jiaotong University School of Medicine, Shanghai, 20032, China; 3 Pathology Center, Shanghai General Hospital/Faculty of Basic Medicine, Shanghai Jiaotong University School of Medicine, No.280, South Chong-Qing Road, Shanghai 200025, People's Republic of China

**Keywords:** integrin β3, PTEN/Akt/mTOR, apoptosis, cardiomyocytes, hypoxia

## Abstract

**Objective**: Integrin β3 is one of the main integrin heterodimer receptors on the surface of cardiac myocytes. Our previous studies showed that hypoxia induces apoptosis and increases integrin β3 expression in cardiomyocytes. However, the exact mechanism by which integrin β3 protects against apoptosis remains unclear. Hence, the present investigation aimed to explore the mechanism of integrin β3 in cardiomyocyte proliferation and hypoxia-induced cardiomyocyte apoptosis.

**Methods**: Stable cells and* in vivo* acute and chronic heart failure rat models were generated to reveal the essential role of integrin β3 in cardiomyocyte proliferation and apoptosis. Western blotting and immunohistochemistry were employed to detect the expression of integrin β3 in the stable cells and rat cardiac tissue. Flow cytometer was used to investigate the role of integrin β3 in hypoxia-induced cardiomyocyte apoptosis. Confocal microscopy was used to detect the localization of integrin β3 and integrin αv in cardiomyocytes.

**Results**: A cobaltous chloride-induced hypoxic microenvironment stimulated cardiomyocyte apoptosis and increased integrin β3 expression in H9C2 cells, AC16 cells, and cardiac tissue from acute and chronic heart failure rats. The overexpression of integrin β3 promoted cardiomyocyte proliferation, whereas silencing integrin β3 expression resulted in decreased cell proliferation *in vitro*. Furthermore, knocking down integrin β3 expression using shRNA or the integrin β3 inhibitor cilengitide exacerbated cobaltous chloride-induced cardiomyocyte apoptosis, whereas overexpression of integrin β3 weakened cobaltous chloride-induced cardiomyocytes apoptosis. We found that integrin β3 promoted cardiomyocytes proliferation through the regulation of the PTEN/Akt/mTOR and ERK1/2 signaling pathways. In addition, we found that knockdown of integrin αv or integrin β1 weakened the effect of integrin β3 in cardiomyocyte proliferation.

**Conclusion**: Our findings revealed the molecular mechanism of the role of integrin β3 in cardiomyocyte proliferation and hypoxia-induced cardiomyocyte apoptosis, providing new insights into the mechanisms underlying myocardial protection.

## Introduction

Heart failure is a major public health concern because of its high mortality, high rate of hospitalization, and cost of management. Heart failure can be categorized into acute heart failure and chronic heart failure. Heart failure leads to hypoxia in cells and tissues. In turn, hypoxia exacerbates the deterioration of heart failure patients, thereby establishing a vicious circle of increasing hypoxia and subsequent malignant progression 1. Therefore, the identification of new diagnostic and therapeutic targets in hypoxia-induced heart failure for improving the prognosis of heart failure patients is required.

Integrins are widely expressed on the cell surface and involve cell-extracellular matrix adhesion and interaction. The integrin family is composed of 24 αβ heterodimeric members. Integrin heterodimers consist of one α and one β subunit 2. Accumulating evidence has shown that the expression of integrins is associated with cell proliferation, migration, invasion, differentiation, and matrix remodeling 3. Integrin β3 is one of the main integrin heterodimer receptors on the surface of cardiomyocytes 4. Extensive studies have shown that integrin β3 contributes to the survival, proliferation and metastatic phenotype of human tumors 2, 5. Previous studies have shown that integrin β3 knockout mice exhibit myocardial cell apoptosis in pressure-overload hypertrophy 6. The overexpression of integrin β3 inhibites lipopolysaccharide (LPS)-induced autophagy in cardiomyocytes 7. Recent studies have shown that integrin β3 is required for the attachment, retention and therapeutic benefits of human cardiospheres in myocardial infarction 8. Therefore, these results suggest that integrin β3 plays an important role in cardiovascular disease. Our previous studies showed that integrin β3 inhibits hypoxia induced apoptosis in cardiomyocytes 9. However, the exact mechanism by which integrin β3 protects against apoptosis remains unclear. In this study, we investigated the exact mechanism of the role of integrin β3 in cardiomyocyte proliferation and hypoxia-induced cardiomyocyte apoptosis.

## Materials and Methods

### Cell lines and cell culture

The rat embryonic cardiomyocyte cell line H9C2 was purchased from the cell bank of the Institute of Biochemistry and Cell Biology of the Chinese Academy of Sciences (Shanghai, China). The human cardiac cell line AC16 was purchased from American Type Culture Collection (ATCC, Rockville, MD). These cell lines were continuously cultured in Dulbecco's modified Eagle's medium (DMEM) containing 10% fetal bovine serum (FBS) at 37°C and 5% CO_2_.

### Primary culture of myocardial cells

Primary rat myocardial cells were isolated according to a previously described protocol 9. Primary rat myocardial cells were isolated from the hearts of 1- to 3-day-old Sprague-Dawley rats with 0.25% trypsin. The cells (1×10^5^ cells/well) were continuously cultured in DMEM containing 10% fetal bovine serum (FBS) at 37°C and 5% CO_2_.

### Plasmids and short hairpin RNAs (shRNAs)

Vectors expressing integrin β3 and control were obtained from Funeng (Guangzhou, China). Short hairpin RNAs (shRNAs) targeting integrin β3 and integrin β1 and a general negative control (NC) shRNA were synthesized by Funeng (Guangzhou, China). shRNA for integrin αv was purchased from GeneChem (Shanghai, China). The fragments were designed to target integrin β3, integrin β1 and integrin αv transcripts. The target sequence of integrin β3 was 5'-GCAAACAACCCATTGTATA-3'. The target sequence of integrin β1-1 was 5'-CCAGAAGACATTACTCAGATC-3'. The target sequence of integrin β1-2 was 5'-CCATACATTAGTACAACACCA-3'. The target sequence of integrin αv was 5'-GATAAGAGGAGTCTCGAGT-3'.

### Lentivirus production and cell transduction

A lentiviral vector (integrin β3), shRNA vectors (shintegrin β3, shintegrin β1 and shintegrin αv) and packaging vector psPAX2 and pMD2G were cotransfected into HEK293T cells using Lipofectamine 2000. The supernatant containing the integrin β3-overexpressing and integrin β3, integrin β1 and integrin αv knockdown lentiviruses was then harvested after 72 hours and filtered through a 0.45-μm sterile syringe. H9C2 and AC16 cells were infected with 1×10^6^ recombinant lentivirus-transducing units in the presence of 6 μg/ml polybrene (Sigma).

### Western blotting

Western blotting was performed according to a previously described protocol 10. The cell pellet was washed with cold PBS once and then lysed with RIPA buffer (Thermo Scientific) containing protease inhibitor, and phosSTOP phosphatase inhibitor cocktail (Roche, Welwyn Garden, Swiss, UK). The lysates were centrifuged at 14,000 rotations per minute for 15 minutes at 4°C, and then the cleared supernatant was collected. The proteins were separated by sodium dodecylsulfate-polyacrylamide gel electrophoresis (SDS-PAGE), electroblotted onto nitrocellulose membranes and probed with primary antibodies. The primary antibodies used for western blotting were against integrin β3 (Abcam, Cambridge, UK), integrin β1 (Abcam, Cambridge, UK), integrin αv (Abcam, Cambridge, UK), phosphor-mTOR (Abcam, Cambridge, UK), mTOR (Abcam, Cambridge, UK), phosphor-ERK1/2 (Cell Signaling Technology, USA), ERK1/2 (Cell Signaling Technology, USA), phosphor-Akt (Cell Signaling Technology, USA), Akt (Cell Signaling Technology, USA), Bcl-2 (Proteintech, China), Bax (Proteintech, China), cleaved caspase 3 (Cell Signaling Technology, USA) , PTEN (Cell Signaling Technology, USA) and β-actin (Sigma, USA). The immunoreactive bands were visualized using an ECL reagent (Pierce, Rockford, IL, USA).

### Quantitative RT-PCR (qRT-PCR)

According to the manufacturer's protocol, total RNA was extracted using TRIzol reagent (Invitrogen, Carlsbad, CA, USA), and cDNA was generated from 1 μg of total RNA using a Prime-Script RT Reagent Kit (TaKaRa, Shanghai, China). qRT-PCR was performed using a 7500 system (Thermo Scientific, MA, USA).The qRT-PCR primer sequences were as follows: integrin β3-F: 5'- AGTCAGCGAGGCCCAGATC-3', integrin β3-R: 5'- AGGGTCTGGATGCTGGACAG-3'; β-actin-F: 5'- AGGCATCCTGACCCTGAAGTAC-3', and β-actin-R: 5'- GAGGCATACAGGGACAACACAG-3'.

### Cell proliferation and colony formation assays

Cell proliferation was detected using a Cell Counting Kit-8 according to the manufacturer's specifications. Colony formation was evaluated by seeding cells at concentrations of 5×10^3^/well in 6-well plates. Once colonies were visible, they were fixed with formaldehyde solution. Then, the cells were stained with Giemsa. The number of colonies per well was counted. Each experiment was performed in triplicate.

### Apoptosis Assay

An Annexin V-AbFluor^TM^ 555 Apoptosis Detection Kit (Abbkine) was used to visualize apoptotic cells according to the manufacturer's instructions. Briefly, 2×10^5^ cells were collected, washed with ice-cold PBS twice and resuspended in 100 μL of Annexin V binding buffer. Next, 4 μL of Annexin V- AbFlourTM 555 was added to the cell suspension and incubated at room temperature for 15 minutes. Next, 400 μL of Annexin V Binding Buffer was added, and the samples were analyzed with a flow cytometer.

### Animal and Treatment

Twenty male Sprague-Dawley rats, aged 8-weeks-old and weighing 220-260 g, were supplied by Jiesijie Laboratory Animal Co. (Shanghai, China) and fed regular food in separate cages at 20-25°C and 60-70% in a humidity-controlled environment. The rats had free access to water that was disinfected with ultraviolet radiation.

Coronary artery ligation was performed as previously described to establish a rat acute myocardial infarction (AMI) model 9. The same procedure without coronary artery ligation was performed in sham controls. All rats were anesthetized and euthanized 6 h after coronary artery ligation.

Chronic heart failure (CHF) model rats were intraperitoneally administered isoproterenol (ISO) (10 mg/kg) or an equal volume of saline daily for two weeks to induce heart failure as previously described. After one month, all rats were sacrificed and euthanized. The hearts were removed, cleared of blood, and immediately transferred to ice-cold containers containing 0.9% sodium chloride.

### Immunohistochemistry (IHC)

Immunohistochemical staining was performed as described previously 9. Paraffin-embedded tissues were sectioned, blocked with 3% H_2_O_2_ in PBS for 15 min at room temperature and then incubated with the indicated antibodies overnight at 4 °C. A horseradish peroxidase (HRP)-conjugated secondary antibody was used, and a DAB kit was employed for signal detection. The control samples were incubated without primary antibody or with normal serum instead of primary antibody.

### Statistical analyses

SPSS 16.0 statistical package (SPSS, Inc., Chicago, IL, USA) or GraphPad Software was used to analyze the data. All results are presented as the mean ± S.D. Two-group comparisons were analyzed using the two-tailed Student's t test. Comparisons of three or more groups were analyzed using one-way ANOVA. Statistical significance was defined as *P < 0.05 and **P < 0.01.

## Results

### The expression of integrin β3 and integrin αv is upregulated in CoCl_2_-induced cardiomyocyte apoptosis

**Figure 1:**
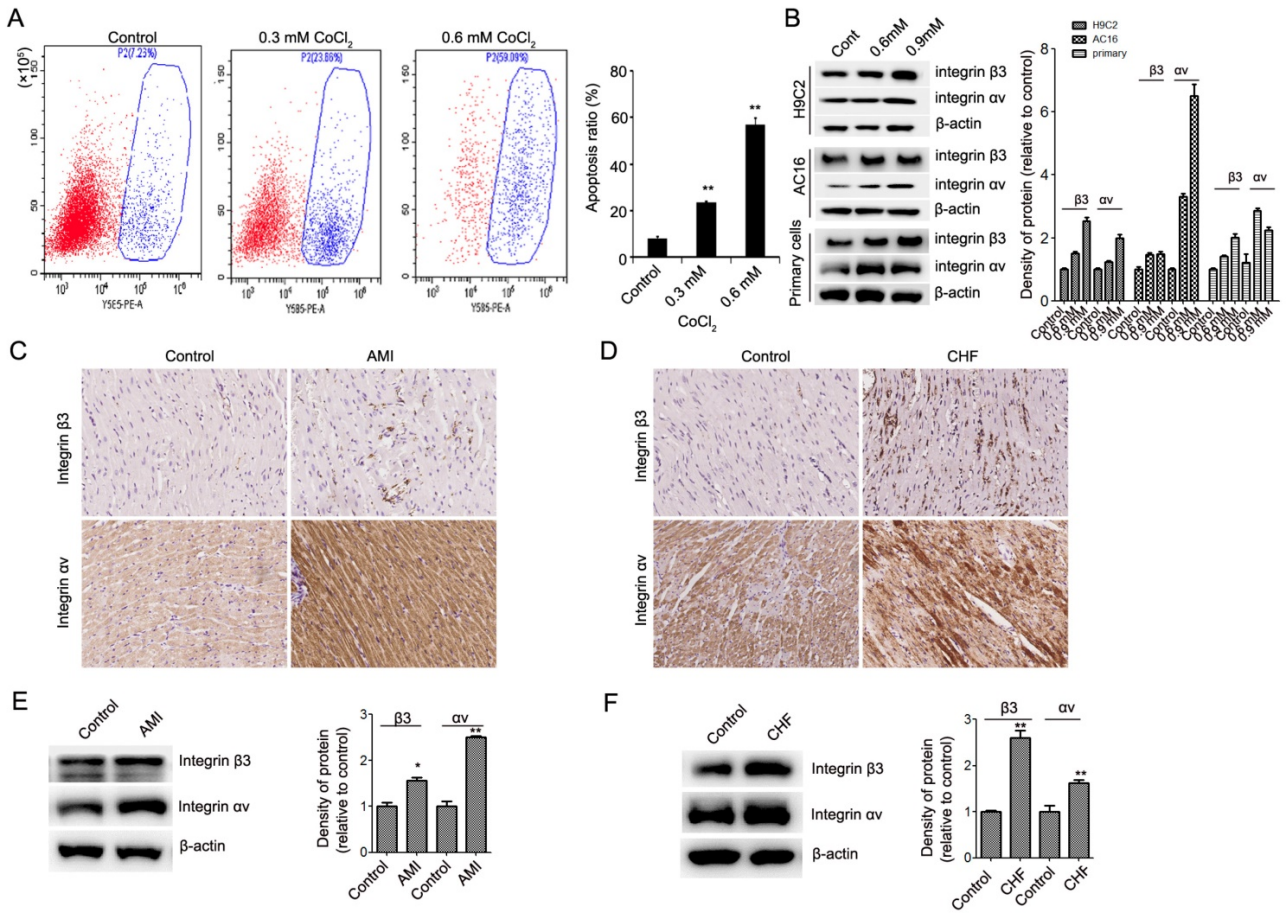
** Expression of integrin β3 and integrin αv in hypoxia-treated cardiomyocytes and myocardial tissue from heart failure rats.** (A) H9C2 cells were treated with CoCl_2_ (600 μM) for 12 hours. Cell apoptosis was detected by flow cytometry. (B) Integrin β3 and integrin αv expression was detected by western blot analysis in H9C2, AC16 and primary rat myocardial cells treated with CoCl_2_. *P<0.05; **P<0.01. (C) Immunohistochemical analysis of integrin β3 and integrin αv in the control and AMI groups. (D) Immunohistochemical analysis of integrin β3 and integrin αv in the control and CHF groups. Representative images are shown (×200). (E) Western blot analysis of integrin β3 and integrin αv in the control and AMI groups. (F) Western blotting analysis of integrin β3 and integrin αv in the control and CHF groups.

We first detected the expression of integrin β3 in CoCl_2_-induced cardiomyocyte apoptosis. Our results showed that CoCl_2_ inhibited cardiomyocyte proliferation and induced cardiomyocyte apoptosis in H9C2, AC16 and primary rat myocardial cells (Figure 1A, Figure S1). We found that the expression of integrin β3 was upregulated in CoCl_2_-induced cardiomyocyte apoptosis (Figure 1B). These results are consistent with our previous finding 9. To confirm the role of integrin β3 in hypoxia-induced cardiomyocyte apoptosis, the expression of integrin β3 was also detected in AMI and CHF rat cardiac tissue. Our IHC results also showed that the expression of integrin β3 was upregulated in AMI and CHF rat cardiac tissue (Figure 1C-1F).

Integrin αv and β3 subunits noncovalently bind to form an integrin αvβ3 heterodimer 12. Therefore, we also detected the expression of integrin αv in hypoxia-induced cardiomyocyte apoptosis. Our results showed that the expression of integrin αv was upregulated in hypoxia-induced cardiomyocytes *in vitro* and *in vivo* (Figure 1B, Figure 1C-1F). These results showed that integrin αvβ3 can play an important role in cardiomyocyte apoptosis.

### Integrin β3 promotes cardiomyocyte proliferation and inhibits CoCl_2_-induced cardiomyocyte apoptosis

**Figure 2:**
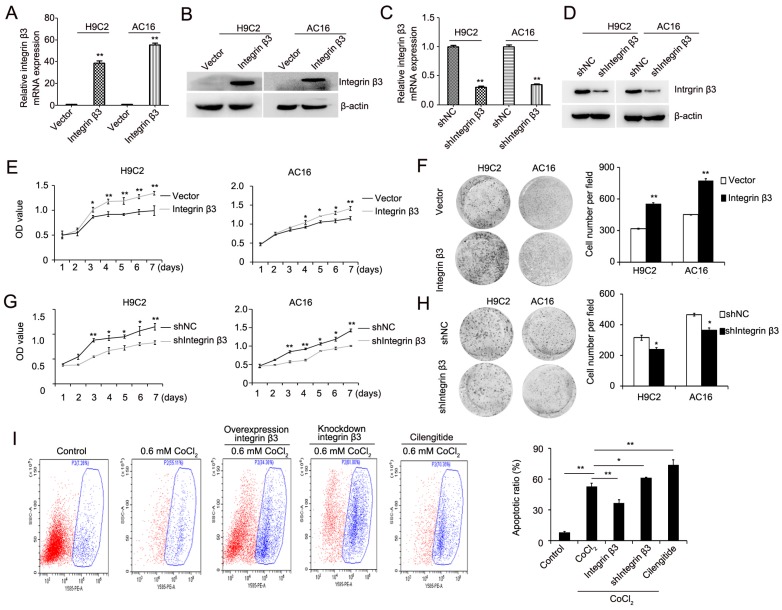
** Integrin β3 increases cardiomyocyte proliferation and weakens CoCl_2_-induced apoptosis.** (A) The mRNA levels of integrin β3 in H9C2 and AC16 cells expressing empty vector or integrin β3 was detected by qRT-PCR. (B) The protein levels of integrin β3 in H9C2 and AC16 cells expressing empty vector or integrin β3 were detected by western blot. (C) The mRNA levels of Integrin β3 in H9C2 and AC16 cells expressing shNC vector or Integrin β3 shRNA was detected by qRT-PCR. (B) The protein levels of integrin β3 in H9C2 and AC16 cells expressing shNC vector or integrin β3 shRNA were detected by western blot. The overexpression of integrin β3 increased cardiomyocyte proliferation (E) and colony-forming ability (F) in H9C2 and AC16 cells. The knockdown of integrin β3 decreased cardiomyocyte proliferation (G) and colony-forming ability (H) in H9C2 and AC16 cells. (I) Integrin β3-overexpressing, integrin β3 knockdown, control and inhibitor cilengitide-treated (10 μM) H9C2 cells were treated with CoCl_2_ (0.6 mM) for 12 h, and apoptosis was detected by flow cytometry. The bar graphs show the quantitative analysis data. *P<0.05; **P<0.05.

We next explored the role of integrin β3 in cardiomyocyte proliferation and apoptosis. Using a lentiviral vector system, we successfully established H9C2 and AC16 cell lines with stable ectopic integrin β3 expression and stable integrin β3 knockdown. The efficiency of the overexpression and knockdown of integrin β3 was verified by qRT-PCR and western blotting (Figure 2A-2D). Our results showed that the overexpression of integrin β3 increased cardiomyocyte proliferation and clone-forming ability (Figure 2E and 2F). Conversely, the knockdown of integrin β3 inhibited cardiomyocyte proliferation and clone-forming ability (Figure 2G and 2H). Therefore, these results suggested that integrin β3 promotes cardiomyocyte proliferation.

**Figure 3:**
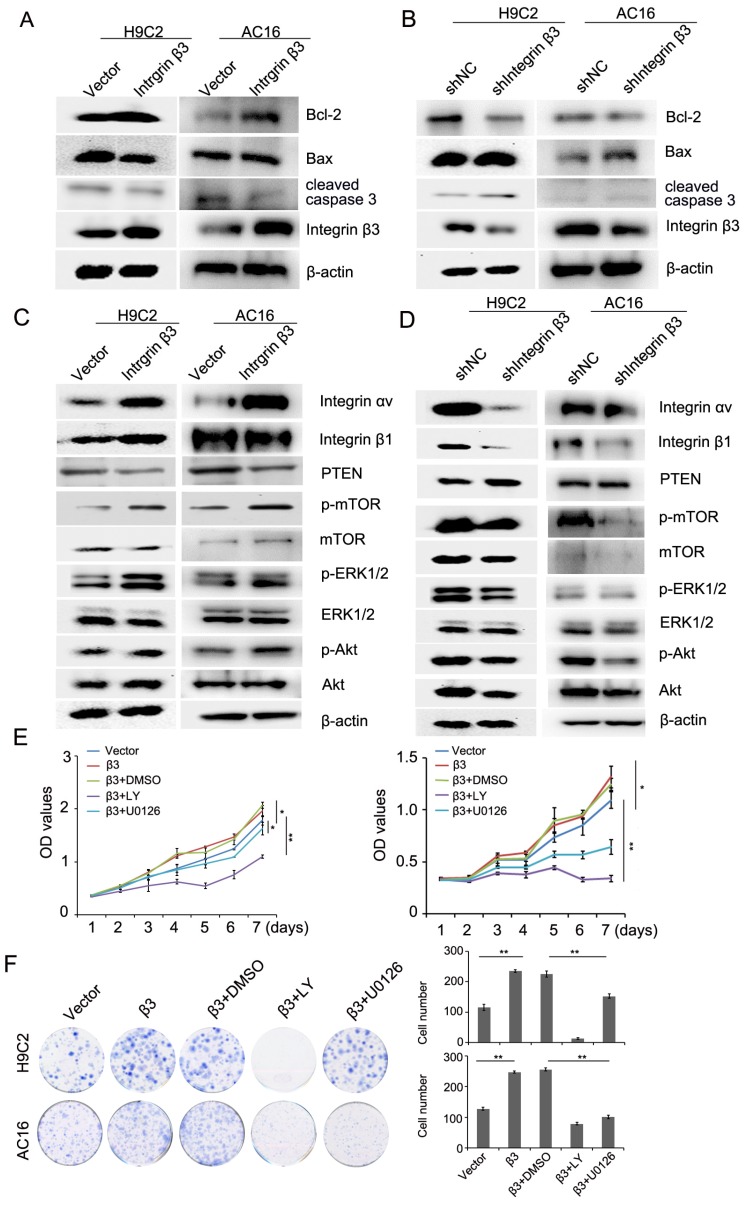
** Integrin β3 promotes cardiomyocyte proliferation through the regulation of the PTEN/Akt/mTOR and ERK1/2 pathways**. (A) Bcl-2, Bax, integrin β3 and cleaved caspase 3 expression was detected by western blotting in integrin β3-overexpressing H9C2 and AC16 cells. (B) Bcl-2, Bax, integrin β3 and cleaved caspase 3 expression was detected by western blotting in integrin β3-knockdown H9C2 and AC16 cells. (C) Integrin β1, integrin αv, PTEN, p-Akt, Akt, p-ERK1/2, ERK1/2, p-mTOR and mTOR were detected by western blotting in integrin β3-overexpressing H9C2 and AC16 cells. (D) Integrin β1, integrin αv, PTEN, p-Akt, Akt, p-ERK1/2, ERK1/2, p-mTOR and mTOR were detected by western blotting in integrin β3-knockdown H9C2 and AC16 cells. (E) Integrin β3-overexpressing H9C2 and AC16 cells were treated with LY294002 (10 μM) and U0126 (10 μM) for the indicated times. Cell proliferation was detected by the CCK8 assay. (F) Integrin β3-overexpressing H9C2 and AC16 cells were treated with LY294002 and U0126 for the indicated times. Cell proliferation was detected by the colony formation assay. *P<0.05; **P<0.01.

We next detected the expression of apoptosis-related proteins during the promotion of cardiomyocyte proliferation by integrin β3. Our results showed that cleaved caspase 3 and pro-apoptotic protein Bax was decreased and that the anti-apoptotic protein Bcl-2 was increased in integrin β3-overexpressing H9C2 and AC16 cells (Figure 3A, Figure S2A). The cleaved caspase 3 and pro-apoptotic protein Bax was increased, and the anti-apoptotic protein Bcl-2 was decreased in integrin β3 knockdown H9C2 and AC16 cells (Figure 3B, Figure S2B). Therefore, these results suggested that integrin β3 promotes cardiomyocyte proliferation and inhibits cardiomyocyte apoptosis.

We also examined the effect of integrin β3 on CoCl_2_-induced cardiomyocyte apoptosis. The results showed that the overexpression of integrin β3 inhibited CoCl_2_-induced cardiomyocyte apoptosis, whereas the knockdown of integrin β3 increased CoCl_2_-induced cardiomyocyte apoptosis (Figure 2I).

**Figure 4:**
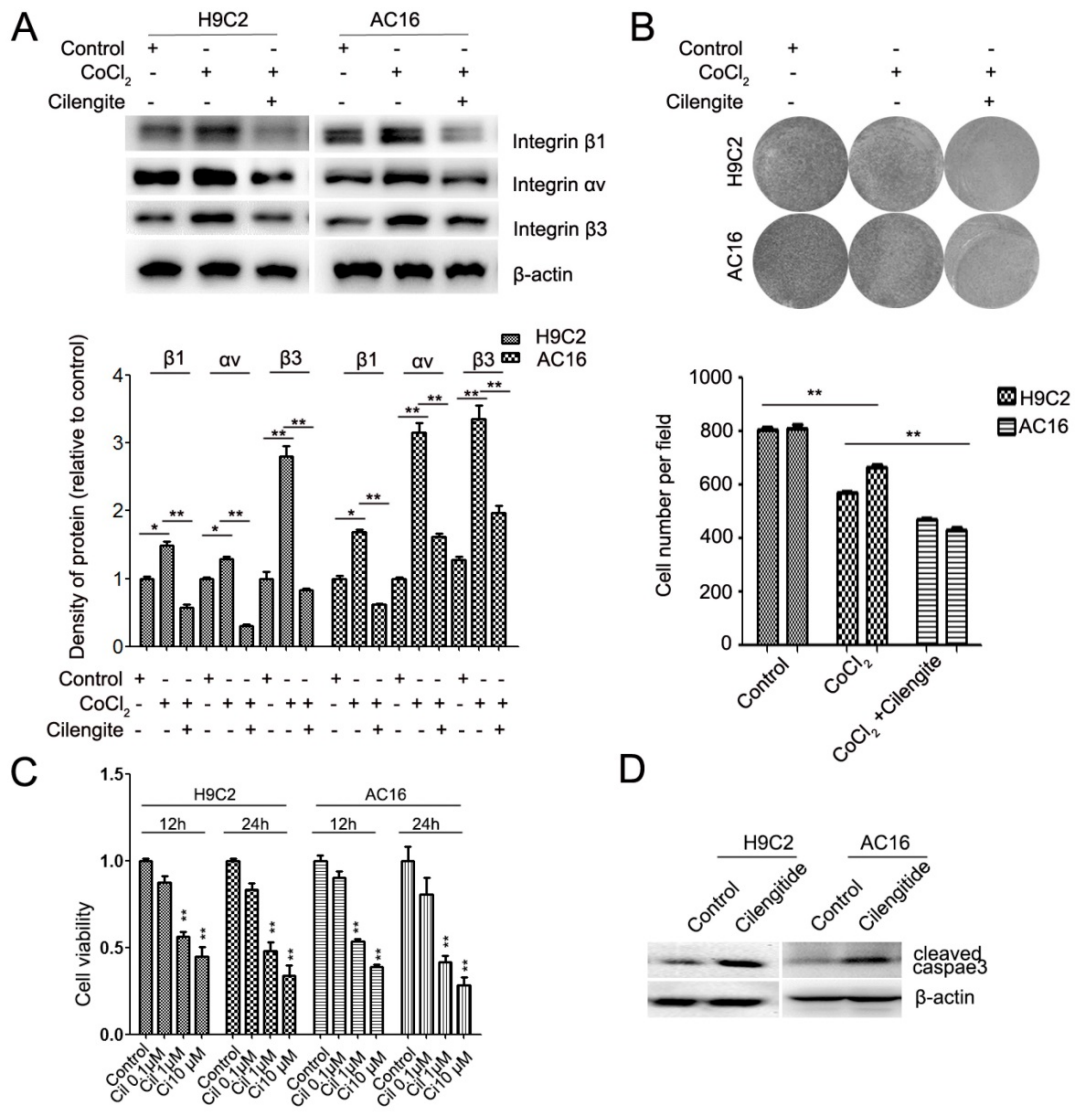
** Cilengitide exacerbates the CoCl_2_-induced inhibition of cardiomyocytes.** (A) The expression of integrin β3, integrin β1 and integrin αv was detected by western blotting in integrin β3-overexpressing H9C2 and AC16 cells. (B) Cilengitide inhibited cardiomyocyte colony-forming ability in CoCl_2_-treated cells. (C) H9C2 and AC16 cells were treated with different dose of cilengitide. Cell viability was detected by the CCk8 assay. (D) The expression of cleaved caspase 3 in H9C2 and AC16 cells treated with cilengitide (10 μM) for 24h. *P<0.05; **P<0.01.

Cilengitide is an integrin αvβ3 receptor antagonist. We detected whether cilengitide exacerbates CoCl_2_-induced cardiomyocyte apoptosis. Our results showed that cilengitide inhibited the expression of integrin αv and β3 in CoCl_2_-treated cells (Figure 4A). Furthermore, cilengitide exacerbated apoptosis and the inhibition of proliferation in CoCl_2_-induced cardiomyocytes (Figure 2I, Figure 4B). In addition, we found that cilengitide inhibited H9C2 and AC16 cells proliferation in a dose-dependent manner. The expression of cleaved caspase 3 was upregulated in the cilengitide-treated cells (Figure 4C and 4D). Therefore, these results showed that integrin β3 promotes cardiomyocyte proliferation and inhibits hypoxia-induced cardiomyocyte apoptosis.

### CoCl_2_ inhibits cardiomyocyte proliferation through the regulation of the PTEN/Akt/mTOR and ERK1/2 signaling pathways

**Figure 5:**
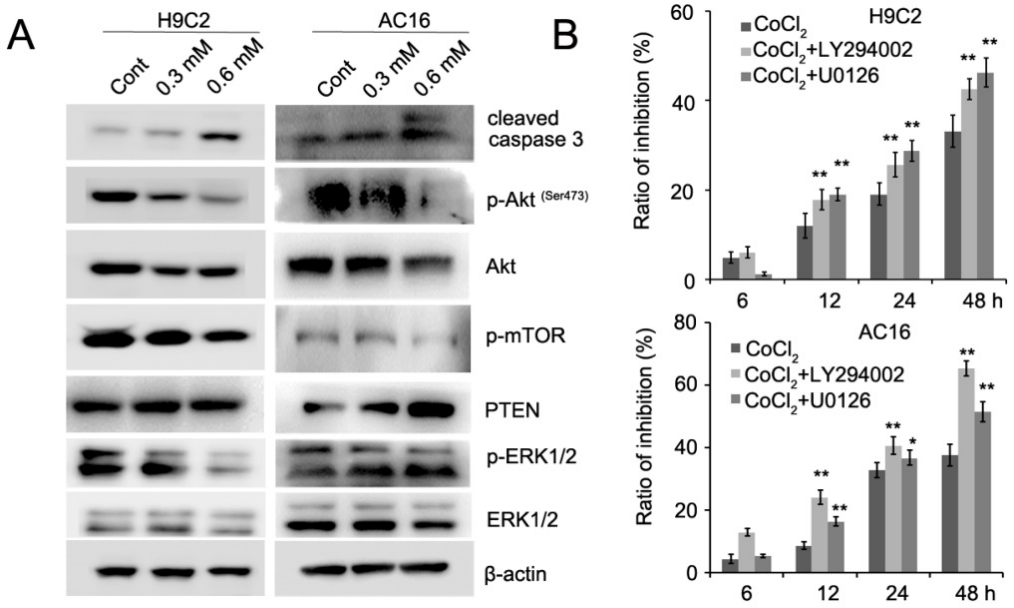
** CoCl_2_ inhibits cardiomyocyte proliferation through the regulation of the PTEN/Akt/mTOR and ERK1/2 pathways.** (A) p-Akt, Akt, p-ERK1/2, ERK1/2, p-mTOR, mTOR and cleaved caspase 3 were detected by western blotting in CoCl_2_-treated H9C2 and AC16 cells. (B) CoCl_2_-treated H9C2 and AC16 cells were treated with LY294002 and U0126 for the indicated times. Cell proliferation was detected by the CCK8 assay. *P<0.05; **P<0.01.

Recent evidence has implicated mTOR as a central regulator of proliferation in various normal and malignant cells 13, 14. Therefore, we detected the expression of mTOR inCoCl_2_-treated cardiomyocytes. Our results showed that CoCl_2_ inhibited mTOR phosphorylation in H9C2 and AC16 cells (Figure 5A). mTOR is activated by Akt which is associated with increased cell proliferation 14. We next detected the expression of Akt phosphorylation in H9C2 and AC16 cells. We found that phosphorylated Akt was decreased and PTEN expression was upregulated in CoCl_2_-treated cells (Figure 5A). In addition, we found that phosphorylated ERK1/2 was decreased in CoCl_2_-treated cardiomyocytes (Figure 5A, Figure S3). To confirm the role of Akt and ERK1/2 in CoCl_2_-treated cardiomyocytes, the Akt inhibitor LY294002 and ERK1/2 inhibitor U0126 were used in CoCl_2_-treated cardiomyocytes. Our results showed that the inhibitory effects of CoCl_2_ on H9C2 and AC16 cells were enhanced by LY294002 and U0126 (Figure 5B). Therefore, these results suggested that the hypoxia-induced inhibition of cardiomyocyte proliferation may be achieved through the PTEN/Akt/mTOR and ERK1/2 signaling pathways.

### Integrin β3 promotes cardiomyocyte proliferation through the regulation of the PTEN/Akt/mTOR and ERK1/2 signaling pathways

The above- mentioned results showed that hypoxia-induced cardiomyocyte apoptosis may be achieved through the PTEN/Akt/mTOR and ERK1/2 signaling pathways. Thus, we hypothesized that integrin β3 may promote proliferation by regulating the PTEN/Akt/mTOR and ERK1/2 signaling pathways. Therefore, we next detected the levels of PTEN/Akt/mTOR and the ERK1/2 signaling pathway. Our results showed that the overexpression of integrin β3 increased the expression of p-Akt, p-ERK1/2, and p-mTOR and inhibited the expression of PTEN (Figure 3C, Figure S2C). Conversely, the knockdown of integrin β3 decreased the expression of p-Akt, p-ERK1/2, p-mTOR and increased expression of PTEN in H9C2 and AC16 cells (Figure 3D, Figure S2D). To confirm these results, we used the Akt inhibitor LY294002 and the ERK1/2 inhibitor U0126 in integrin β3 overexpressing H9C2 and AC16 cells. Our results showed that LY294002 and U0126 suppressed proliferation and clone-forming ability (Figure 3E and 3F). Therefore, these results indicated that integrin β3 promotes cardiomyocyte proliferation through regulation of the PTEN/Akt/mTOR and ERK1/2 signaling pathways.

### Integrin αv and integrin β1 coordinate with integrin β3 to mediate cardiomyocyte proliferation

**Figure 6:**
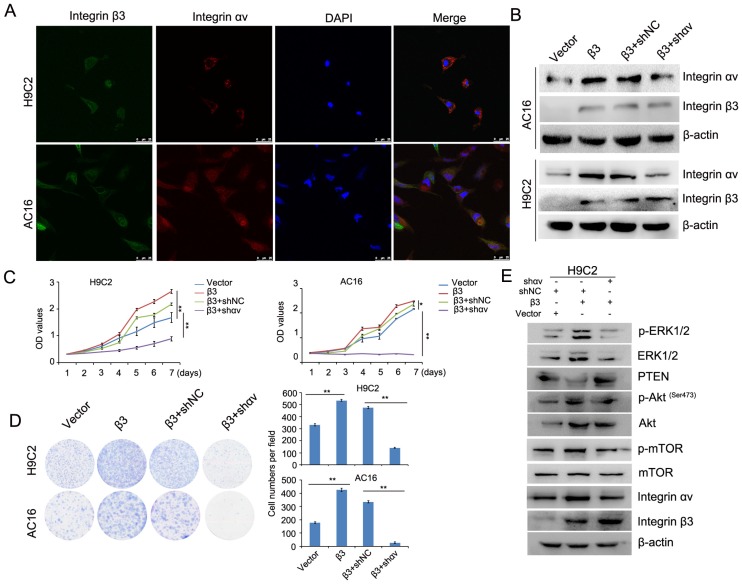
** Knockdown of integrin αv weakens the effect of integrin β3 on cardiomyocyte proliferation and clone-forming ability in H9C2 and AC16 cells.** (A) Confocal microscopy was used to detect the localization of integrin β3 and integrin αv in H9C2 and AC16 cells. (B) Integrin β3-overexpressing H9C2 and AC16 cells were transfected with integrin αv shRNA as indicated, and the expression of integrin β1 and integrin β3 was detected by western blotting. Cell proliferation (C) and colony-forming ability (D) were measured by the CCK8 assay and colony formation assay. (E) Integrin β3-overexpressing H9C2 cells were transfected with integrin αv shRNA as indicated, and the expression of integrin β3, integrin αv, mTOR, p-mTOR, Akt, p-Akt, PTEN, ERK1/2 and p-ERk1/2 were detected by western blotting.*P<0.05, **P<0.01.

A previous report showed that integrin β1 and integrin β3 can bind to integrin αv to form heteromers 12. We found that the expression of integrin β1 and integrin αv was upregulated in integrin β3-overexpressing cardiomyocytes. The expression of integrin β1 and integrin αv was decreased in integrin β3 knockdown cardiomyocytes. Therefore, we speculated whether integrin β1 and integrin αv play an important role in integrin β3-induced cardiomyocyte proliferation. Furthermore, the colocalization of integrin β3 and integrin αv was found in cardiomyocytes using confocal microscopy (Figure 6A). Next, we used shRNA to decrease the expression of integrin αv in integrin β3 overexpressing cardiomyocytes (Figure 6B). The results showed that silencing integrin αv weakened the effect of integrin β3 on cardiomyocyte proliferation and clone-forming ability (Figure 6C and 6D). Furthermore, the expression of p-Akt, p-ERK1/2 and p-mTOR was slightly inhibited in integrin αv knockdown cardiomyocytes (Figure 6E).

**Figure 7:**
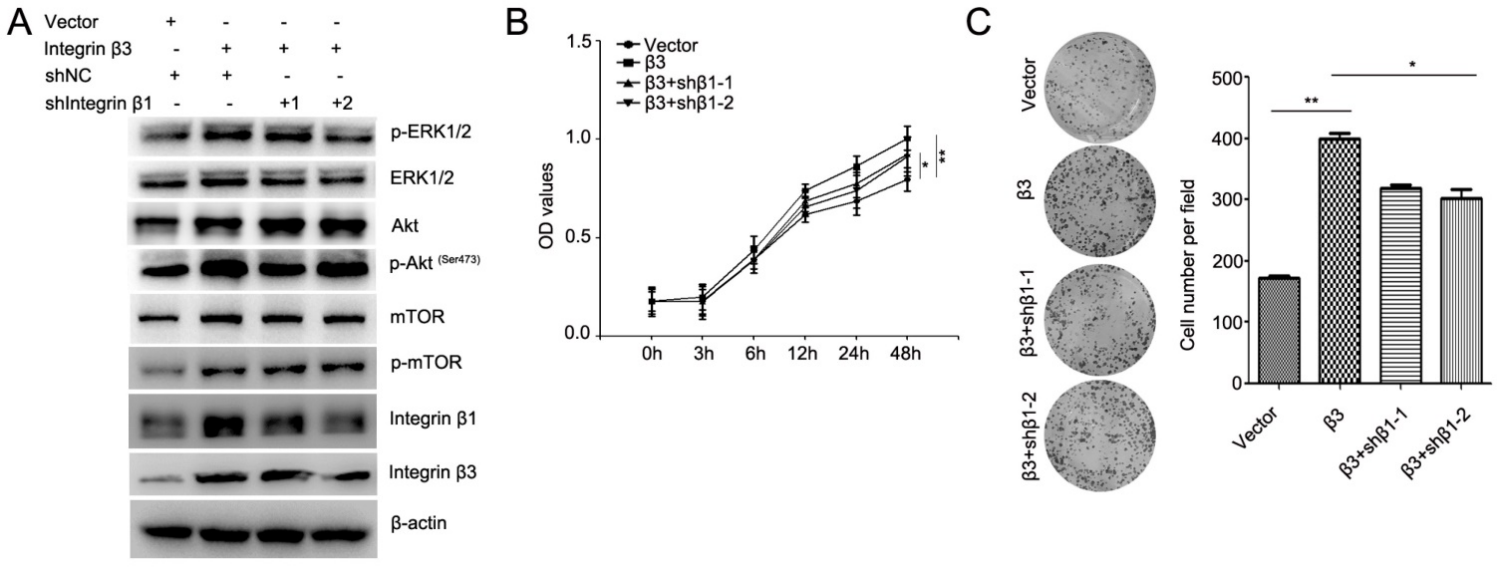
** Knockdown of integrin β1 weakens the effect of integrin β3 on cardiomyocyte proliferation and clone-forming ability in H9C2 cells.** (A) Integrin β3-overexpressing H9C2 cells were transfected with integrin β1 shRNA as indicated, and the expression of p-Akt, Akt, p-ERK1/2, ERK1/2, p-mTOR, mTOR, integrin β1 and integrin β1 were detected by western blotting. Cell proliferation (B) and colony-forming ability (C) was measured by the CCK8 assay and colony formation assay. *P<0.05, **P<0.01.

To confirm the role of integrin β1 in integrin β3-mediated cardiomyocyte proliferation, the expression of integrin β1 was inhibited using two integrin β1 shRNAs (Figure 7A, Figure S4). Our results showed that knockdown of integrin β1 weakened the effect of integrin β3 on cardiomyocyte proliferation and clone-forming ability in H9C2 and AC16 cells (Figure 7B and 7C). Meanwhile, the expression of p-Akt, p-ERK1/2 and p-mTOR was slightly inhibited in integrin β1 knockdown cardiomyocytes (Figure 7A). Therefore, we speculated that integrin β1 and integrin αv coordinates with integrin β3 to mediate cardiomyocyte proliferation.

## Discussion

In the present study, we found that the expression of integrin β3 was upregulated in hypoxia-induced cardiomyocyte apoptosis *in vitro* and *in vivo*. The overexpression of integrin β3 inhibited CoCl_2_-induced cardiomyocyte apoptosis, whereas knocking down integrin β3 expression using shRNA or the integrin β3 inhibitor cilengitide exacerbates cobaltous chloride-induced cardiomyocyte apoptosis. Furthermore, we found that integrin β3 promotes cardiomyocyte proliferation through the regulation of the PTEN/Akt/mTOR and ERK1/2 signaling pathways. In addition, we found that integrin β1 coordinated with integrin β3 to mediate cardiomyocytes proliferation. Thus, the results of the present study demonstrated the mechanism by which integrin β3 protects against cardiomyocyte apoptosis.

Integrin β3, a well-known member of the integrin family, has been studied extensively in cell proliferation and metastasis in various cancers 15, 16. Recent studies have shown that integrin β3 plays an important role in cardiovascular diseases. Liu *et al* reported that inhibiting integrin β3 reduces the attachment, retention and therapeutic benefits of human cardiospheres in mice with acute myocardial infarction 8. Misra *et al.* reported that enhanced integrin β3 signaling is a crucial link between elastin deficiency and arterial hypermuscularization and that integrin β3 blockade is a promising and much needed noninvasive therapeutic approach for supravalvular aortic stenosis 17. Integrin β3 knockout mice develop mild cardiac hypertrophy and inflammation 18. In this study, we found that integrin β3 was upregualted in AMI and CHF rat myocardial tissues. The results are consistent with previous reports 9, 19. In addition, knocking down integrin β3 also increases apoptosis in cardiomyocytes treated with hydrogen peroxide 20. Therefore, these results suggested that integrin β3 plays a protective role in cardiomyocyte apoptosis.

Akt is known as a serine/threonine kinase that plays a vital role in the regulation of cell survival, proliferation, angiogenesis, and metabolism 21. Our data demonstrated that the expression of integrin β3 is upregulated in CoCl_2_-treated cardiomyocytes and that this upregulation is accompanied by the downregulation of phosphorylated Akt. Furthermore, we found that the overexpression of integrin β3 increased phosphorylated Akt, whereas knockdown of integrin β3 decreased phosphorylated Akt in H9C2 and AC16 cells. Therefore, integrin β3 promotes cardiomyocyte proliferation through regulation of Akt phosphorylation. Following activation, Akt directly activates mammalian target of rapamycin complex 1 (mTORC1) by phosphorylating mTORC1 at Ser2448 22. In addition, PTEN negatively controls Akt activation, resulting in decreased recruitment of Akt to the cell membrane 21. Our results also showed that the overexpression of integrin β3 activated phosphorylated mTOR and inhibited PTEN expression. Furthermore, similar results were also observed in AMI and CHF rat models. Johnston *et al*. used integrin β3 knockout mice to demonstrate that integrin β3 is critical for activating NF-κB-mediated cell survival signaling in cardiomyocytes 23. NF-κB is a downstream component of the PI3K/Akt pathway, and is activated by PI3K/Akt pathway through the phosphorylation of IκB kinase (IKK), leading to IκB degradation 24. Therefore, we consider that integrin β3 increases cardiomyocyte proliferation through the regulation of PTEN/Akt/mTOR pathway, leading to NF-κB activation.

The ERK1/2 signaling pathway also plays animportant role in the regulation of cell survival and proliferation. We also found that the overexpression of integrin β3 increases ERK1/2 phosphorylation in cardiomyocytes. Previous studies have shown that crosstalk may occur between the PI3K and ERK1/2 pathways 25, 26. Therefore, we speculate that integrin β3 promotes cardiomyocyte proliferation through the regulation of PTEN/Akt/mTOR and ERK1/2 signaling pathways.

Precious studies have shown that integrin β1 and integrin β3 can bind to integrin αv to form heteromers 12. In this study, we found that the overexpression of integrin β3 increases integrin β1 expression, whereas the knockdown of integrin β3 inhibited integrin β1 expression. To exclude the role of integrin β1 in integrin β3-induced cell proliferation, integrin β1 shRNA was used during integrin β3-induced cell proliferation. We found that knockdown of integrin β1 weakened the effect of integrin β3 on cardiomyocyte proliferation and clone-forming ability in H9C2 and AC16 cells. Therefore, we believe that integrin β1 coordinates with integrin β3 to mediate cardiomyocyte proliferation.

## Supplementary Material

Supplementary figures.

## References

[1] Rochette L, Malka G, Cottin Y (2017). Hypoxia and heart regeneration: A new paradoxical approach for cardioprotection. Arch Cardiovasc Dis.

[2] Cooper J, Giancotti FG (2019). Integrin Signaling in Cancer: Mechanotransduction, Stemness, Epithelial Plasticity, and Therapeutic Resistance. Cancer Cell.

[3] Moreno-Layseca P, Icha J, Hamidi H (2019). Integrin trafficking in cells and tissues. Nat Cell Biol.

[4] Harston RK, Kuppuswamy D (2011). Integrins are the necessary links to hypertrophic growth in cardiomyocytes. J Signal Transduct.

[5] Noh KW, Sohn I, Song JY (2018). Integrin beta3 Inhibition Enhances the Antitumor Activity of ALK Inhibitor in ALK-Rearranged NSCLC. Clin Cancer Res.

[6] Suryakumar G, Kasiganesan H, Balasubramanian S (2010). Lack of beta3 integrin signaling contributes to calpain-mediated myocardial cell loss in pressure-overloaded myocardium. J Cardiovasc Pharmacol.

[7] Zhu Y, Li L, Gong S (2015). β3-integrin inhibits lipopolysaccharide-induced autophagy in cardiomyocytes via the Akt signaling pathway. Cardiology.

[8] Liu S, Jiang Z, Qiao L (2018). Integrin beta-3 is required for the attachment, retention and therapeutic benefits of human cardiospheres in myocardial infarction. J Cell Mol Med.

[9] Su Y, Tian H, Wei L (2018). Integrin beta3 inhibits hypoxia-induced apoptosis in cardiomyocytes. Acta Biochim Biophys Sin (Shanghai).

[10] Jiang Z, Zhou Q, Ge C (2019). Rpn10 promotes tumor progression by regulating hypoxia-inducible factor 1 alpha through the PTEN/Akt signaling pathway in hepatocellular carcinoma. Cancer Lett.

[11] Tian H, Ge C, Zhao F (2017). Downregulation of AZGP1 by Ikaros and histone deacetylase promotes tumor progression through the PTEN/Akt and CD44s pathways in hepatocellular carcinoma. Carcinogenesis.

[12] Cai W, Chen X (2006). Anti-angiogenic cancer therapy based on integrin alphavbeta3 antagonism. Anticancer Agents Med Chem.

[13] Martelli AM, Buontempo F, McCubrey JA (2018). Drug discovery targeting the mTOR pathway. Clin Sci (Lond).

[14] Janku F, Yap TA, Meric-Bernstam F (2018). Targeting the PI3K pathway in cancer: are we making headway?. Nat Rev Clin Oncol.

[15] Wen S, Hou Y, Fu L (2019). Cancer-associated fibroblast (CAF)-derived IL32 promotes breast cancer cell invasion and metastasis via integrin beta3-p38 MAPK signalling. Cancer Lett.

[16] Parvani JG, Gujrati MD, Mack MA (2015). Silencing beta3 Integrin by Targeted ECO/siRNA Nanoparticles Inhibits EMT and Metastasis of Triple-Negative Breast Cancer. Cancer Res.

[17] Misra A, Sheikh AQ, Kumar A (2016). Integrin beta3 inhibition is a therapeutic strategy for supravalvular aortic stenosis. J Exp Med.

[18] Ren J, Avery J, Zhao H (2007). Beta3 integrin deficiency promotes cardiac hypertrophy and inflammation. J Mol Cell Cardiol.

[19] Sun M, Opavsky MA, Stewart DJ (2003). Temporal response and localization of integrins beta1 and beta3 in the heart after myocardial infarction: regulation by cytokines. Circulation.

[20] Shewchuk LJ, Bryan S, Ulanova M (2010). Integrin beta3 prevents apoptosis of HL-1 cardiomyocytes under conditions of oxidative stress. Can J Physiol Pharmacol.

[21] Noorolyai S, Shajari N, Baghbani E (2019). The relation between PI3K/AKT signalling pathway and cancer. Gene.

[22] Hennessy BT, Smith DL, Ram PT (2005). Exploiting the PI3K/AKT pathway for cancer drug discovery. Nat Rev Drug Discov.

[23] Johnston RK, Balasubramanian S, Kasiganesan H (2009). Beta3 integrin-mediated ubiquitination activates survival signaling during myocardial hypertrophy. FASEB J.

[24] Yang L, Hu X, Mo YY (2019). Acidosis promotes tumorigenesis by activating AKT/NF-kappaB signaling. Cancer Metastasis Rev.

[25] Aksamitiene E, Kiyatkin A, Kholodenko BN (2012). Cross-talk between mitogenic Ras/MAPK and survival PI3K/Akt pathways: a fine balance. Biochem Soc Trans.

[26] Dent P (2014). Crosstalk between ERK, AKT, and cell survival. Cancer Biol Ther.

