# Supplementary material for: Integrin β3 promotes cardiomyocyte proliferation and attenuates hypoxia-induced apoptosis via regulating the PTEN/Akt/mTOR and ERK1/2 pathways
Source: Int J Biol Sci. 2020 Jan 14;16(4):644–54. doi: 10.7150/ijbs.39414 (PMC6990915; doi:10.7150/ijbs.39414)
Supplement: Supplementary file 1 — Supplementary figures. [file ijbsv16p0644s1.pdf]

Integrin β3 promotes cardiomyocyte proliferation and attenuates hypoxia-induced apoptosis via regulating the PTEN/Akt/mTOR and ERK1/2 pathways

Lijiang Wei[1], Qingqing Zhou[2], Hua Tian[2]*, Yifan Su[1], Guo-hui Fu[3], Ting Sun[1]*

[1]Department of Cardiology, Shanghai Ninth People's Hospital, Shanghai Jiaotong University School of Medicine. Shanghai, 200025, China

[2]State Key Laboratory of Oncogenes and Related Genes, Shanghai Cancer Institute, Renji Hospital, Shanghai Jiaotong University School of Medicine, Shanghai, 20032, China

[3]Pathology Center, Shanghai General Hospital/Faculty of Basic Medicine, Shanghai Jiaotong University School of Medicine, No.280, South Chong-Qing Road, Shanghai 200025, People's Republic of China

Correspondence address: Ting Sun, MD, Department of Cardiology, Shanghai Ninth People's Hospital Affiliated with Shanghai Jiaotong University School of Medicine, Shanghai 200025, China. Tel/Fax: +86-86-21-23271287; E-mail: beibeisun2008@163.com

Hua Tian, MD, State Key Laboratory of Oncogenes and Related Genes, Shanghai Cancer Institute, Renji Hospital, Shanghai Jiaotong University School of Medicine, 25/Ln 2200, Xietu Road, Shanghai 200032, China. Tel/Fax: +86-21-64432140; E-mail: htian@shsci.org

Running title: Integrin β3 promotes cardiomyocyte proliferation

Supplementary data

Supplementary Figures

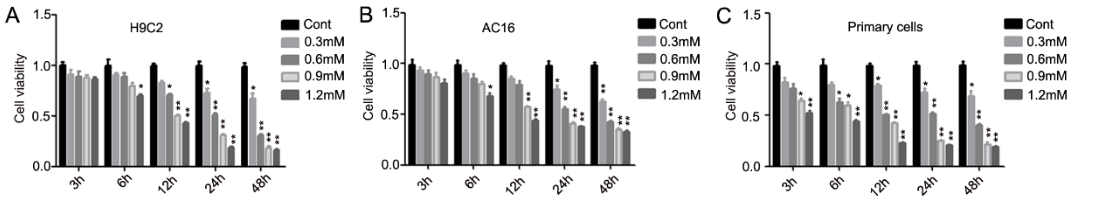

Figure S1. $CoCl_2$ inhibited cardiomyocytes proliferation. H9C2 (A), AC16 (B) and primary ratmyocardial cells (C) were treated with $CoCl_2$. Cell viability was detected by CCk8 assay.

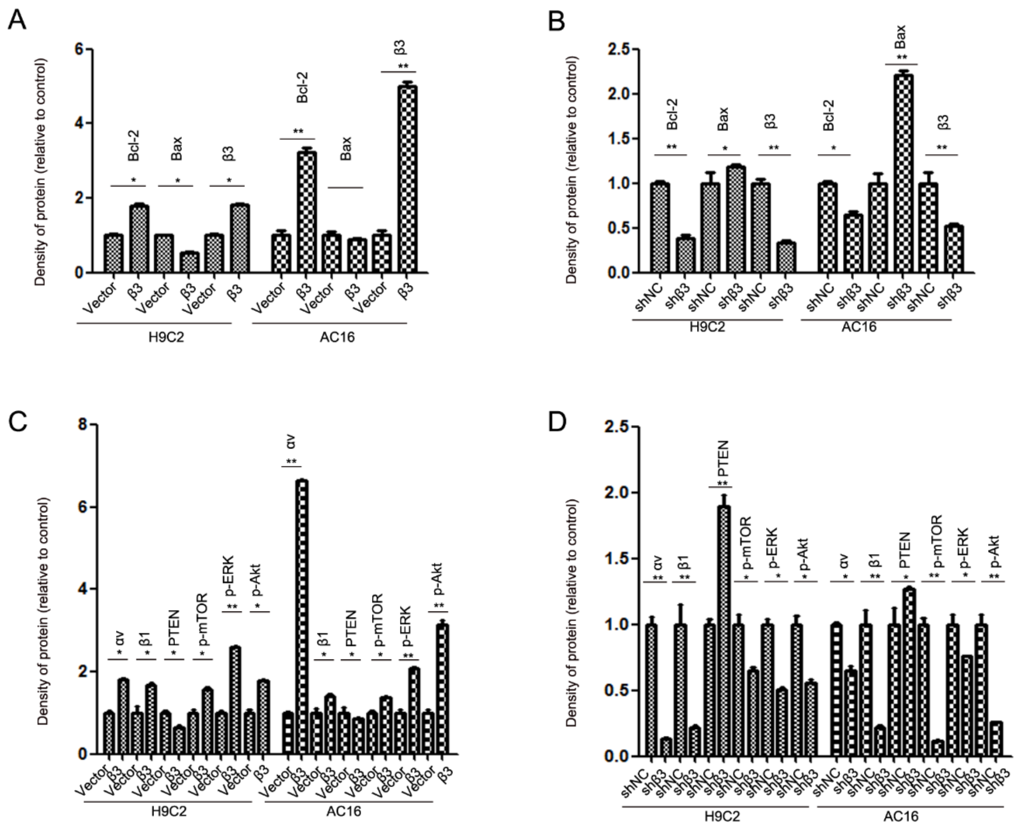

Figure S2.Integrin β3 promotes cardiomyocytes proliferation through regulation of PTEN/Akt/mTOR and ERK1/2 pathway. (A)Protein expression level of Figure 3A. (B) Protein expression level of Figure 3B. (C) Protein expression level of Figure 3C. (D) Protein expression level of Figure 3D.*P<0.05; **P<0.01.

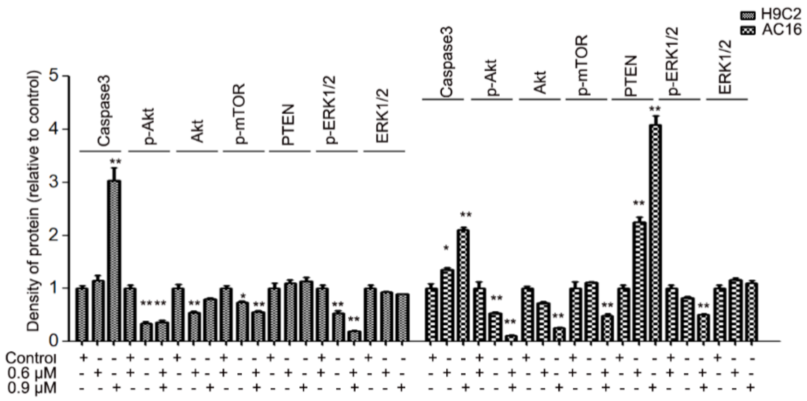

Figure S3.**$CoCl_2$ inhibited cardiomyocytes proliferation through regulation of PTEN/Akt/mTOR and ERK1/2 pathway.** Protein expression level of Figure 5A. *P<0.05; **P<0.01.

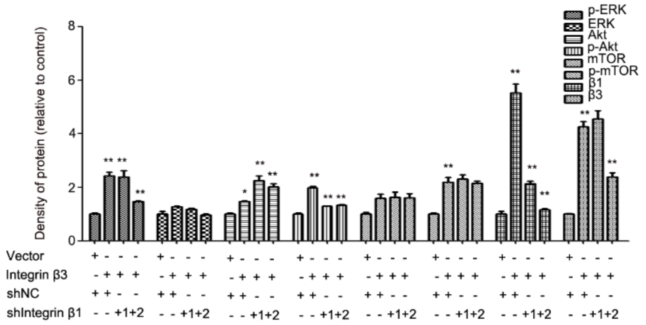

Figure S4.**Knockdown of integrin β1 weaken the effect of integrin β3 on cardiomyocytes proliferation and clone-forming ability in H9C2 cells**.Protein expression level of Figure 7A. *$P<0.05$; **$P<0.01$.
